# A Unique Case of Disseminated Cutaneous Coccidioidomycosis Years After Initial Infection

**DOI:** 10.7759/cureus.80033

**Published:** 2025-03-04

**Authors:** Mason Seely, Christina W Sun, Christopher Smith, Nicole R Bender

**Affiliations:** 1 Internal Medicine, University of Florida College of Medicine, Gainesville, USA; 2 Dermatopathology, University of Florida College of Medicine, Gainesville, USA; 3 Dermatology, Flatirons Dermatology, Broomfield, USA; 4 Dermatopathology, Sagis Diagnostics, Houston, USA

**Keywords:** cutaneous coccidioides, dermatopathology, granulomatous disease, pseudoepitheliomatous hyperplasia, valley river fever

## Abstract

Coccidioides is a dimorphic fungus that causes coccidioidomycosis, also known as San Joaquin Valley Fever. The fungus is endemic to the southwestern United States, northern Mexico, and Central and South America. Infection is typically acquired through inhalation of dust particles that causes pulmonary disease. Direct cutaneous inoculation can occur but is rare.

Histopathological examination of cutaneous lesions typically shows pseudoepitheliomatous hyperplasia with acute, suppurative granulomatous inflammation. Fungal organisms tend to be found more superficially and vary in density. Although non-caseating and sarcoidal granulomas have been described in late lesions of coccidioidomycosis, these tend to be in the upper two-thirds of the dermis.

In this report, we describe a case of disseminated cutaneous coccidioidomycosis in a 21-year-old female seen as a dermatology outpatient for scar revision with unique histopathological findings including deep dermal non-caseating granulomas and absence of pseudoepitheliomatous hyperplasia. With further investigation, a remote history of self-resolving Valley Fever years ago was revealed confirming the diagnosis of coccidioidomycosis. This case is unusual due to the delay in presentation from the patient’s original infection and atypical histopathologic findings.

## Introduction

Coccidioides is a dimorphic fungus that causes coccidioidomycosis, also known as San Joaquin Valley Fever. It is caused by dimorphic species of *Coccidioides immitis* and *Coccidioides posadasii* endemic to the southwestern United States, northern Mexico, and Central and South America [1]. Infection is typically acquired through inhalation of dust particles that causes pulmonary disease. Occasionally, patients with pulmonary disease can have hematogenous or lymphatic spread to the bone, brain, and/or skin with systemic disease occurring in 1% of immunocompromised hosts [1-3]. Primary cutaneous disease, caused by direct inoculation or exposure at sites of trauma, is rare with less than 100 cases reported in the literature [4-6]. Clinical presentation of primary cutaneous coccidioidomycosis is variable with most cases reported describing non-ulcerated nodules that appear after a 2-3-week inoculation period [4,5]. 

On histological examination, cutaneous lesions of coccidioidomycosis typically show pseudoepitheliomatous hyperplasia, a reactive epidermal thickening seen in many infections, with acute, suppurative granulomatous inflammation [4,7]. Fungal organisms tend to be found more superficially and vary in density [4-6]. Hematoxylin and eosin (H&E), periodic acid-Schiff (PAS), and Gomori methenamine silver (GMS) stains are all useful in the examination of granulomas and help reveal spherules [1]. We report a case of cutaneous coccidioidomycosis with unusual histologic findings.

## Case presentation

**Figure 1:**
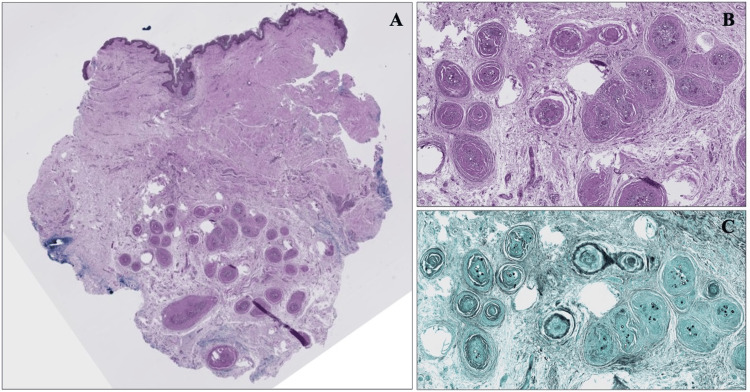
A. Scanning magnification with H&E stain demonstrating epithelioid granulomas in the deep dermis and subcutis beneath a dermal scar. B. High-power magnification of epithelioid granulomas. C. GMS stain highlighting thick-walled spherules within granulomas and multinucleated giant cells. The fungal structures appear as clustered black spherules. H&E, hematoxylin and eosin; GMS, Gomori methenamine silver.

**Figure 2:**
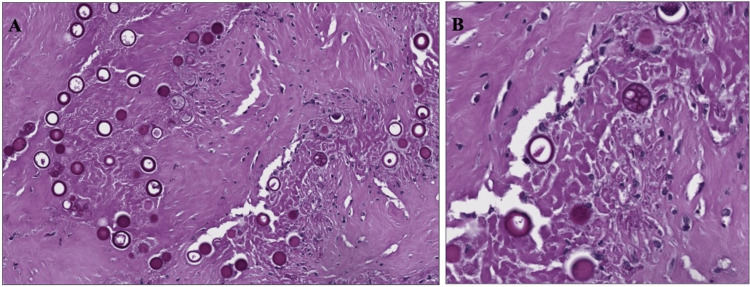
A. Thick-walled spherules, 20-40 micrometers in diameter, with B. PAS-positive endospores. PAS, periodic acid-Schiff.

A healthy 21-year-old female from southern California presented to an outpatient dermatology clinic for a scar revision at the site of a previously excised draining lymph node secondary to systemic Valley Fever over 10 years ago. At her prior visit, she did not receive treatment aside from node excision and her symptoms self-resolved. She did not have a history of immunocompromise or other past medical history. At her presentation to dermatology, the scar was excised from the right inferolateral neck and sent for histopathological testing. H&E stains revealed non-caseating granulomas with spherules in the deep dermis and subcutis beneath a dermal scar (Figure 1). GMS stains and PAS stains were then applied highlighting these thick-walled spherules, predominantly 20-40 micrometers in diameter, some of which were filled with PAS-positive endospores, within the granulomas and multinucleated giant cells (see Figure 1 and Figure 2). Fite-Faraco and Ziehl-Neelson stains were negative for acid-fast bacilli. Uniquely, this patient had deep dermal granulomas, an absence of pseudoepitheliomatous hyperplasia, and a remote nature of prior infection. The pathology results were reviewed with the patient at a follow-up appointment. After discussing the findings and potential implications including indolent inoculation versus latent systemic infection, further medical therapy was deferred given the remote nature of her prior infection and lack of systemic symptoms.

## Discussion

Coccidioidomycosis infection causes symptoms in approximately 40% of patients including cough, angina, fever, and erythema nodosum. Disseminated cutaneous infection can also occur. Histopathology of early lesions is characterized by dense inflammatory infiltrates composed of neutrophils, lymphocytes, and plasma cells [7]. Later in the disease process, granulomas and epithelioid inflammation develop, which typically contain double-walled spherules 10-80 micrometers in diameter filled with endospores [6,7].

Diagnosis is often made through a combination of detailed clinical history, serology, culture, and histopathology. In this report, we document a case of cutaneous coccidioidomycosis with unique histomorphology diagnosed several years after the initial infection. It is important to recognize that both acute and chronic signs of inflammation can be found in the infected tissues. The presence of neutrophils and eosinophils clustered around ruptured spherules suggests active disease while chronic granulomatous inflammation around unruptured spherules is more commonly found in patients with controlled disease [3,5-7]. Our specimen shows sarcoidal granulomas with organisms in the deep dermis and subcutis without overlying epidermal changes such as pseudoepitheliomatous hyperplasia to suggest an underlying infection. Additionally, lymphatic invasion may be another reason that overlying epidermal changes are not seen. Therefore, clinicians should consider a deeper biopsy if the index of suspicion for cutaneous coccidioidomycosis is high. Although suppurative granulomatous dermatitis is the most common pattern seen in cutaneous infections, sarcoidal granulomas should also prompt further workup for infection in the appropriate clinical context. When coccidioidomycosis is suspected, thorough clinical history remains paramount with a history of prior residence or recent travel to an endemic area remaining critical for diagnosis.

In this patient's case, with known prior history of coccidioidomycosis infection and lymph node removal, it is also plausible that direct inoculation occurred at the time of the prior surgery with scar revision revealing a site of indolent inoculation rather than a latent product of systemic infection. At the time of prior diagnosis and lymph node excision, treatment was deferred. In patients with known risk factors like immunocompromisation or with more moderate disease, treatment with antifungal agents including fluconazole and itraconazole can be given for a duration of 3-6 months [3].

## Conclusions

Cutaneous coccidioidomycosis is a rare clinical entity that typically exhibits pseudoepitheliomatous hyperplasia with acute, suppurative granulomatous inflammation surrounding clusters of spherules and endospores. In this report, we document a case of cutaneous coccidioidomycosis with unique histomorphology including deep dermal granulomas and the absence of pseudoepitheliomatous hyperplasia diagnosed several years after initial infection. When coccidioidomycosis is suspected, detailed clinical history remains paramount to diagnosis and treatment can be initiated depending on patient symptoms and risk factors. A deeper biopsy should also be considered so that pathology is not missed.
